# Does chlorhexidine improve periodontal health and bacterial profiles in patients with special health care needs? A systematic review and meta-analysis

**DOI:** 10.3389/froh.2025.1656328

**Published:** 2025-11-18

**Authors:** Deepak Sharma, Han-Pang Liu, Yung-Ting Hsu, Fatima Sheyda, Anna Forsyth, Travis Nelson

**Affiliations:** 1 Department of Pediatric Dentistry, University of Washington, Seattle, WA, United States; 2 Private Practitioner, Taichung, Taiwan; 3 Department of Periodontics, University of Washington, Seattle, WA, United States; 4 University of Washington, Seattle, WA, United States

**Keywords:** chlorhexidine, evidence based dentistry, oral hygiene, periodontal index, intellectual disability, developmental disabilities

## Abstract

**Objectives:**

The purpose of this systematic review was to evaluate the effectiveness of chlorhexidine (CHX)-containing products as adjuncts to mechanical oral hygiene practices in maintaining gingival health in patients with intellectual disability.

**Materials and methods:**

An electronic search was conducted in three databases—PubMed, Embase, and Web of Science—1945- December 31, 2024. Two calibrated independent reviewers assessed the selected studies based on the inclusion and exclusion criteria. The main outcomes measured were changes in the gingival index, plaque index, and complications. A meta-analysis was performed to analyze the efficiency of adjunct CHX products compared with controls (mechanical plaque removal only). Additionally, meta-regression was conducted to investigate the factors contributing to these outcomes.

**Results:**

Twelve randomized controlled trials involving individuals with special health care needs (SHCN) were included across varied clinical settings. CHX use was associated with a statistically significant reduction in plaque accumulation (Hedges' g = −1.491; 95% CI: −2.067 to −0.914; *P* < .001; I^2^ = 37.3%), with the greatest reductions observed in studies using spray and gel delivery methods. Gingival inflammation also decreased significantly across studies (mean difference = −0.214; 95% CI: −0.306 to −0.121; *P* < .001), with 0.2% CHX formulations demonstrating the most consistent improvement.

**Conclusion:**

In patients with SHCN short–term use (4–6 weeks), especially with 0.2% formulations, appeared to offer the greatest benefit while maintaining acceptable tolerability. While adverse effects such as tooth staining and taste alterations are common, they are generally mild and self-limiting. These findings support the short-term use of CHX as an adjunctive measure in oral hygiene management for individuals with SHCN, with use tailored to the patient's needs and monitored closely by a dental provider to minimize complications.

**Systematic Review Registration:**

(CRD420251003198).

## 1 Introduction

An estimated 1.3 billion people worldwide are currently living with disabilities. Of these, 107.6 million (1.39% of the global population) have intellectual disability (ID) (1). While poor oral outcomes can be prevented by maintaining good oral hygiene and regular professional examination and cleaning, the oral health of patients with of ID lags that of people without ID (2). This is largely due to physical and mental challenges that interfere with regular plaque control (3). Therefore, the American Academy of Pediatric Dentistry (AAPD) defines Special Health Care Needs (SHCN) as, “any physical, developmental, mental, sensory, behavioral, cognitive, or emotional difference or limiting condition that requires medical management, health care intervention, and/or use of specialized services or programs. The condition may be congenital, developmental, or acquired through disease, trauma, or environmental cause and may impose limitations in performing daily self-maintenance activities or substantial limitations in a major life activity.” (4) This broad definition encompasses both mental and physical differences that impact health and daily living. As a result, chemotherapeutic agents such as chlorhexidine (CHX) have been used in an attempt to reduce plaque accumulation and improve periodontal health in this population.

A recent systematic review found that CHX reduced plaque and gingivitis compared with placebo and other controls in the general population. However, CHX use resulted in a large increase in tooth staining and other side-effects such as taste disturbance, burning sensation, soreness, and tissue irritation (5). Other research has also shown that antibacterial mouth rinses can alter nitric oxide metabolism, increasing blood pressure and predisposing patients to metabolic disorders, including diabetes (6–8). While the most recent systematic review on this topic did not specifically assess the use of CHX in patients with SHCN, the authors suggested that extended use of CHX mouthrinse in SHCN individuals must be carefully weighed against the adverse effects associated with its use (5).

While the efficacy of CHX has been well established in the general population, its effects and potential impacts on the oral health of individuals with SHCN have not been systematically examined. To address this gap, we conducted a systematic review and meta-analysis to investigate these outcomes in this specific patient population. The primary purpose of this systematic review was to evaluate the effectiveness of CHX-containing products as adjuncts to mechanical oral hygiene practices in maintaining gingival health in pediatric and adult patients with SHCN. The secondary goal was to assess side effects of CHX, including changes in oral bacterial profile, in this patient population. Given the substantial challenges that this high-risk population endures with mechanical plaque removal and their susceptibility to periodontal disease, this review aims to assist clinicians by synthesizing the current evidence and supporting clinical decision-making for safe and effective CHX use.

## 2 Materials and methods

This systematic review was conducted following the PRIMSA (Preferred Reporting Items for Systematic Reviews and Meta-analyses) guideline (9), and registered on Prospero (registration number: CRD420251003198). Ethical approval was not required for this review.

### 2.1 Search strategy

A literature search was performed across three electronic databases: PubMed, Embase, and Web of Science. The articles selected were published from January 1, 1945 to December 31, 2024. The search strategy is detailed in Supplementary Appendix 1.

#### 2.1.1 Focused questions

Based on the PICO principle, we prepared our focused questions:

In patients with SHCN(P), does CHX (I), compared to control patients receiving placebo or mechanical plaque removal only (C), change the following outcomes (O), including periodontal parameters, bacterial profiles, and lead to any complications?

The primary outcomes in this review were gingival index (GI) and plaque index (PI) as periodontal clinical measurements. Secondary outcomes included complications/side effects of CHX use, including changes in the oral bacterial profile.

In the current review, we applied the AAPD definition of SHCN uniformly, however there was variation regarding the mental diagnosis of participants due to changes in diagnostic criteria for mental disorders over the nearly 40-year period in which the studies were conducted. According to the Diagnostic and Statistical Manual of Mental Disorders, 2nd edition (DSM-II, 1968–1980), mental retardation (MR) was defined as significantly subaverage intellectual functioning (10). DSM-III added the requirement of limitations in adaptive behavior (1980–1987) (11), and DSM-IV (1994–2013) required that these impairments must be accompanied by limitations in adaptive functioning in at least two domains (e.g., communication, self-care, social skills) (12). The *DSM-5* (2013–present) replaced the term MR with “intellectual disability (intellectual developmental disorder),” (ID) which is now more widely used in clinical and educational contexts (13).

#### 2.1.2 Study selection criteria

##### 2.1.2.1 Inclusion criteria

1. Studies published in English
2. Randomized controlled trials (RCTs) evaluating the periodontal impacts of CHX in the oral cavity
3. Studies enrolling >10 patients in each group
4. Study population was patients with ID or SHCN

##### 2.1.2.2 Exclusion criteria

1. *in vitro* studies, case reports, animal studies, retrospective studies, narrative reviews, unpublished data, communications, or expert opinions
2. Studies in which the intervention included chemotherapeutic agents other than CHX

#### 2.1.3 Information sources and search strategy

In applying the eligibility criteria, two trained and calibrated researchers (DS and FS) worked independently and addressed any discrepancies through consensus. The selection process was conducted in two stages. To identify studies meeting inclusion criteria, all titles and abstracts derived from the search strategy were screened. Subsequently, the full texts were reviewed to verify inclusion criteria. Any disagreements between the reviewers were resolved by discussing with a third reviewer (X).

The following information was extracted: study ID, authors, year, study design, control, number of participants, sex, age, smoker or non-smoker, CHX concentration, CHX product type, CHX application frequency, mechanical plaque removal methods, GI baseline, GI postoperative, PI baseline, PI postoperative, bleeding index (BI) baseline, BI postoperative, probing depth (PD) baseline, PD postoperative, oral microflora (OM) baseline, OM postoperative, follow-up period, and main findings.

### 2.2 Quality assessment

Version 2 of the Cochrane risk-of-bias tool for randomized trials (RoB2) (14) was used to evaluate the quality of RCTs. The assessment was independently performed by two examiners (X and X). The disagreement was later resolved by discussion with the third person (X) as needed.

### 2.3 Grading the body of evidence

The Grading of Recommendations Assessment, Development and Evaluation (GRADE) system was used to assess the quality of evidence. Several factors were considered, including risk of bias in reported outcomes, inconsistency among study outcomes, indirectness of outcome reporting, imprecision in reported outcomes, and potential publication bias. The GRADE system categorizes evidence quality into four levels: high, moderate, low, and very low. Evidence quality is evaluated based on five criteria: risk of bias, inconsistency, indirectness, imprecision, and publication bias.

### 2.4 Data synthesis

Two independent authors (X and X) extracted data from the included studies. When multiple post-treatment time points were reported, data from the end of the intervention were used for analysis (15). For crossover studies, we included only the first study interval to avoid carry-over effects if the paired-analysis was not available (15). The random-effects model assumes that the true effect could vary from study to study, owing to variability in study populations, it was employed using Comprehensive Meta-Analysis software (version 3; Biostat) with a significance level of *α* = 0.05 (16). Mean differences, and 95% confidence intervals (CIs) were calculated for gingival indices. Studies lacking pre- and post-intervention means and standard deviations were excluded from the quantitative analysis, except for Russell 1978, which provided mean differences and their standard deviations, allowing calculation by the software program. In outcomes related to plaque reduction, inconsistence in plaque indices was noted. Hedges' *g* was selected as the primary effect size estimate due to its comparative accuracy with Cohen's d and Glass' *Δ*. Effect sizes were interpreted as small (0.2), moderate (0.5), or large (0.8) (17).

Heterogeneity was assessed using the I^2^ statistic and categorized as low (25%), moderate (50%), or high (75%) (18). Subgroup analyses were conducted based on CHX concentration and product type. Meta-regression was performed to evaluate whether treatment duration influenced CHX's effect on gingival index reduction. Sensitivity analyses, using a one-study removal approach, assessed the robustness of the findings (19). Publication bias was examined according to Cochrane Handbook guidelines and funnel plots were generated and visually assessed (20).

## 3 Results

A total of 5,551 records were identified through database searches. After removing 1,751 duplicate records (1,729 via Covidence and 22 manually), 3,800 titles and abstracts were screened. Following full-text screening, 12 studies were selected for review and qualitative synthesis. No ongoing studies or studies awaiting classification were identified. The PRISMA flowchart detailing the selection process is provided in Supplementary Appendix 2. Excluded studies are detailed in Supplementary Appendix 3.

### 3.1 Characteristics of selected papers

#### 3.1.1 Characteristics of trial design and settings

Table 1 shows an overview of the key characteristics of all selected articles. A total of 12 RCTs, including 560 patients in the CHX group and 575 controls, were included in the qualitative synthesis. All studies were RCTs conducted in institutional, community-based, hospital, school, or home settings across diverse geographic regions (21–32).

**Table 1:** Overall characteristics of included studies.

Study	Design; setting; country	Patients (M/F); age	Study Duration	CHX product	CHX concentration (percent)	CHX frequency	Comparator	Outcome (PI, GI, PD, CI, OM)
Pannuti et al. (2003)	RCT; Institution; Brazil	43 (27/16); 24.6 years mean age	8 weeks	Gel	0.5%	2x/day	Placebo gel	PI, GI
Chibinski et al. (2011)	RCT; at-home; Brazil	29 (16/13); 7–12 years	Four 10-day experiment periods with 15-day washout intervals	Gel & Spray	0.12%	3x/day	Placebo gel & spray	PI, GI
Kalaga et al. (1989)	RCT; Adult training center; UK	47 (23/24); 21–59 years *45 of 47 successfully completed the study.	Two 31-day experiment periods with a 30-day washout interval	Spray	0.20%	2x/day	Placebo spray	PI, BI, PD
Lotufo et al. (2003)	RCT; Hospital; Brazil	30 (18/12); 17–35 years	16-week study (experiment period included 8 weeks of gel use)	Gel	0.5%	2x/day	Placebo gel	PI, OM
Laher and Cleaton (1995)	RCT; School; South Africa	153 (86/67); 6–21 years *129 of 153 succesfully completed the study.	Two 2-week experiment periods with 8-week washout interval	Mouthrinse	0.2%	2x/day	Placebo rinse	PI, GI
Cutress et al. (1977)	RCT; Institution; New Zealand	117 (n/a); 10–20 years	6 months	Gel	1.0%	1x/day	Placebo gel	PI, GI, CI
Chikte et al. (1991)	RCT; Hospital; South Africa	52 (n/a); 10–26 years	9 weeks (3-week experiment period, 3-week washout interval)	Spray	0.2%	2x/day	Placebo spray	PI, GI, OM
Viana et al. (2014)	RCT; Institution; Brazil	26 (13/13/); 7–14 years	2 months	Spray	0.12%	2x/day	Placebo spray	PI, GI, BI
Russell et al. (1978)	RCT; Hospital; Denmark	30 (19/11) 3–14 years *29 of 30 successfully complete the study.	7 months (Two 2-month experiment periods, with a minimum 2-month washout interval)	Gel	1.0%	1x/day	Placebo gel	PI, GI
Bay et al. (1975)	RCT; Institution; Denmark	54 (34/20) 7–14 years	Two 6-week experiment periods with an 8-week washout interval	Mouthrinse	0.2% first period, then 0.1% second period	2x/day	Placebo mouthrinse	PI, GI
Stiefel et al. (1992)	RCT; rehabilitation settings; Denmark	76 (46/30); 40.8 mean age	Two 10-week experiment periods, separated by a 6-week washout interval	Mouthrinse (applied via cotton swab)	0.12%	1x/day	Placebo swab	PI, GI, CI, PD
Gallagher et al. (1977)	RCT; Institution; New Zealand	117 (n/a); 10–20 years	6 months	Gel	1.0%	1x/day	Placebo gel	OM

PI, plaque index; GI, gingival index; PD, probing depth; CI, calculus index; IM, oral microflora; BI, bleeding index. *Provides additional information about sample size.

Four studies were conducted in Brazil (21, 25, 27, 28). Three studies were conducted in Denmark (22, 23, 31). Two studies were conducted in South Africa (24, 30). Two studies were conducted in New Zealand (29, 32). One study was conducted in the United Kingdom (26).

#### 3.1.2 Characteristics of participants

Participants ranged in age from 3 to 59 years, encompassing both pediatric and adult populations with SHCN. Sample sizes varied from 26 to 153 participants.

Eight studies (Pannuti et al., 2003; Lotufo et al., 2003; Cutress et al., 1977; Chikte et al., 1991; Viana et al., 2014; Bay et al., 1975; Stiefel et al., 1992; Gallagher et al., 1977) focused on individuals with ID. Two studies (Kalaga et al., 1989; Russell et al., 1978) included participants with both mental and physical limitations. One study (Laher & Cleaton, 1995) mentioned only physical disabilities with mental status unclear, and one (Chibinski et al., 2011) broadly described participants as children with “special needs” without specifying the nature of the disabilities.

#### 3.1.3 Characteristics of interventions

The various formulations of CHX, included mouthrinses (23, 24, 31), sprays (21, 25, 26, 30), and gels. (22, 25, 27–29, 32) Concentrations ranged from 0.12% to 1.0%, with dosing frequencies of one to three times daily and intervention durations ranging from 4 weeks to 7 months. Most studies employed twice-daily administration (21, 24, 26–28, 30, 31). Eleven studies (21, 22, 24–32), included two study arms, while one study (23) included three (Table 1).

#### 3.1.4 Characteristics of comparators and outcomes

Control groups primarily received either mechanical oral hygiene alone, or placebo formulations matched the mode of CHX delivery. Table 1 summarized the characteristics of comparators. The primary outcomes assed were PI and GI (Tables 2, 3, 4). The secondary outcome of OM was reported in section 3.2.3.

**Table 2:** Meta-analysis summary table.

Outcome	Numbers of studies	Effect estimate
Plaque index	6 studies (7 data sets)	Hedges’ *g* = −1.491 95% CI: −2.067 to −0.914; *P* < .001
Gingival index	4 studies	Mean difference = –0.214 95% CI: –0.306 to –0.121; *P* < .001

**Table 3:** Summary table for subgroup analysis of plaque index.

Outcome		Numbers of studies	Effect estimate
CHX concentration	0.12%	3 studies	Hedges’ g = –1.772 (95% CI: –2.764 to –0.779; 95% PI: –4.554 to 1.011; *P* < .001)
	0.2%	2 studies	Hedges’ g = –1.300 (95% CI: –2.661 to –0.060; 95% PI: –4.339 to 1.738; *P* = .061)
	1%	1 study	Hedges’ g = –0.900 (95% CI: –2.849 to 1.050; 95% PI: –4.448 to 2.648; *P* = .366)
CHX Product type	Spray	1 study	Hedges’ g = –1.935 (95% CI: –2.844 to –1.025; 95% PI: –4.151 to 0.282; *P* < .001)
	Rinse	2 studies	Hedges’ g = –0.742 (95% CI: –2.210 to 0.726; 95% PI: –3.425 to 1.941; *P* = .322)
	Topical application	3 studies	Hedges’ g = –1.347 (95% CI: –2.224 to –0.470; 95% PI: –3.541 to 0.847; *P* = .003)

**Table 4:** Summary table for subgroup analysis of gingival index.

Outcome		Numbers of studies	Effect estimate
CHX concentration	0.12%	2 studies	MD = −0.167 (95% CI: −0.430 to 0.096; 95% PI: −0.992 to 0.658; *P* = .212)
	0.2%	1 study	MD = −0.300 (95% CI: −0.598 to −0.002; 95% PI: −1.181 to 0.581; *P* = .048)
	1%	1 study	MD = −0.160 (95% CI: −0.444 to 0.124; 95% PI: −1.018 to 0.698; *P* = .269)
CHX Product type	Spray	1 study	MD = −0.167 (95% CI: −0.430 to 0.096; *P* = .212)
	Rinse	1 study	MD = −0.300 (95% CI: −0.429 to 0.171; *P* < .001)
	Topical application	2 studies	MD = −0.183 (95% CI: −0.261 to 0.106; *P* < .001)

### 3.2 Outcomes

#### 3.2.1 Plaque Index

Eleven of the 12 studies reported PI outcomes in 1,135 patients (560 in test group and 575 in controls) (1–3, 6–14, 21–31). The most frequently used index was the Löe and Silness PI (*n* = 6) (22–24, 26, 29, 30). The other indices being used were the Quigley and Hein Index (*n* = 1) (25), the Simplified Oral Hygiene Index by Greene and Vermillion, (*n* = 1) (21) and the O'Leary Plaque Control Record. (*n* = 1) (27) The remaining studies (31, 32) did not specify the index used. These indices allowed for standardized plaque assessment, despite some methodological differences.

Among these, the Quigley and Hein index was used in one study and the Simplified Oral Hygiene Index (OHI-S) was used in another (21, 25). The remaining 6 studies employed the PI by Silness and Löe (21–26). One study included multiple arms and was treated as two distinct study units, resulting in a total of seven data sets (25).

**Figure 1:**
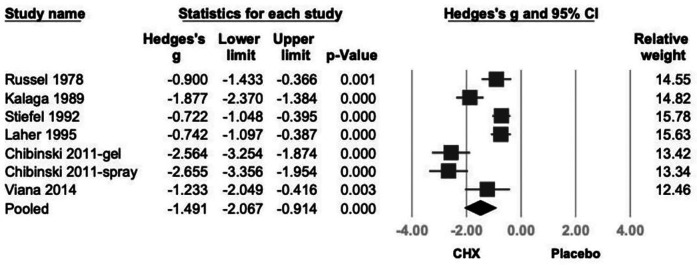
Forest plot of the effects of CHX on PI as compared with the placebo. CHX was found to be effective in reducing PI. CHX, chlorhexidine; CI, confidence interval; PI, plaque index.

CHX was associated with a statistically significant reduction in PI (Hedges' *g* = −1.491; 95% CI: −2.067 to −0.914; 95% PI: −3.493 to 0.512; *P* < .001), although substantial heterogeneity was observed. (*I*^2^ = 89.0%) (Figure 1).

**Figure 2:**
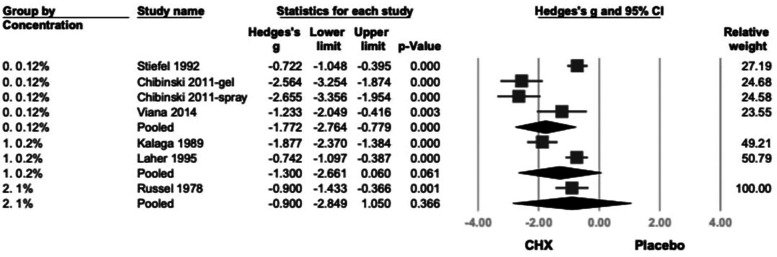
The forest plot of subgroup analysis using concentration of products as the moderator, including 0.12%, 0.2%, and 1 %. CHX is significantly effective in reducing PI in the 0.12% and 0.2% subgroups. CHX, chlorhexidine; CI, confidence interval; PI, plaque index.

Subgroup analysis based on CHX concentration classified studies into 0.12%, 0.2%, and 1% (21–26). A statistically significant reduction in PI was observed in the 0.12% and 0.2% (Hedges' *g* = −1.772; 95% CI: −2.764 to −0.779; 95% PI: −4.554 to 1.011; *P* < .001) subgroup. Although 1% CHX group showed similar trends, they did not reach statistical significance compared to controls. (0.2%: Hedges' *g* = −1.300; 95% CI: −2.661 to −0.060; 95% PI: −4.339 to 1.738; *P* = .061, 1%: Hedges' *g* = −0.900; 95% CI: −2.849 to 1.050; 95% PI: −4.448 to 2.648; *P* = .366) (Figure 2).

**Figure 3:**
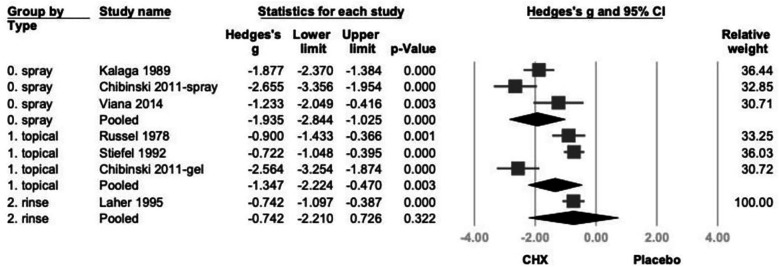
The forest plot of subgroup analysis using the type of products as the moderator, including spray, topical, and rinse. CHX is significantly effective in reducing PI in the spray and topical subgroup. CHX, chlorhexidine; CI, confidence interval; PI, plaque index.

Further subgroup analysis based on product type categorized studies into spray, rinse, and topical application (swabs, gauzes, or toothpaste) (21–26). CHX showed statistically significant effects in both spray (Hedges' *g* = −1.935; 95% CI: −2.844 to −1.025; 95% PI: −4.151 to 0.282;*P* < .001) and topical (Hedges’ *g* = −1.347; 95% CI: −2.224 to −0.470; 95% PI: −3.541 to 0.847; *P* = .003) subgroups. While the rinse subgroup showed a similar direction of effect, the result was not statistically significant. (Hedges' *g* = −0.742; 95% CI: −2.210 to 0.726; 95% PI: −3.425 to 1.941; *P* = .322) (Figure 3).

**Figure 4:**
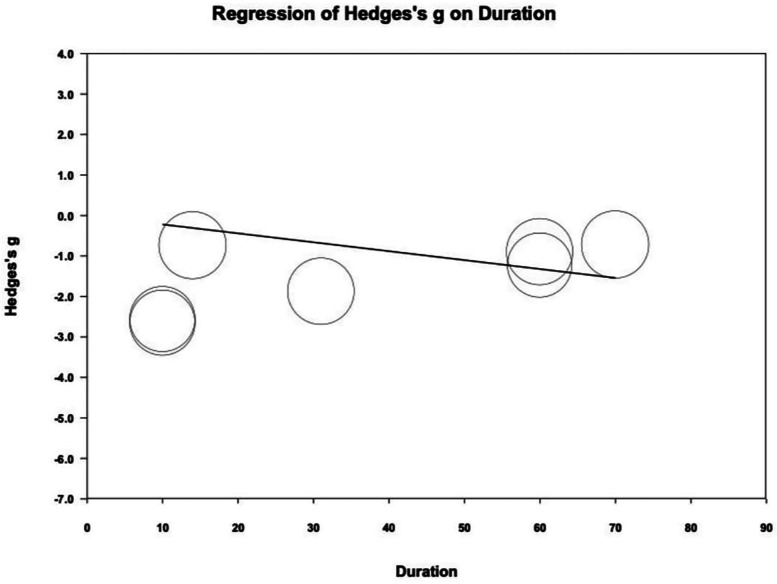
Meta-regression of Hedges's g on treatment duration (day). The coefficient was -0.0220 with statistical significance (*P* = 0.042).

Meta-regression analysis indicated a statistically significant correlation between treatment duration and PI reduction. (coefficient = −0.0220 per day; *P* = .042) (Figure 4). Funnel plot inspection revealed some asymmetry, Egger's regression test showed a *P*-value of 0.049, suggesting potential publication bias (Supplementary Appendix 4).

#### 3.2.2 Gingival Index

**Figure 5:**
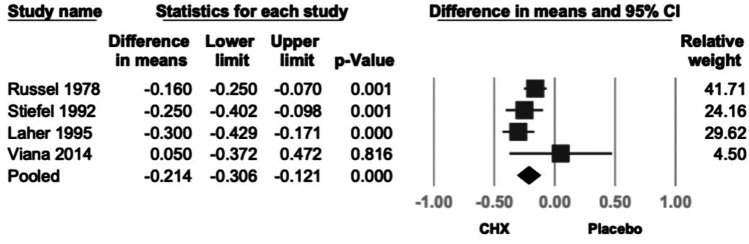
Forest plot of the effects of CHX on GI as compared with the placebo. CHX was found to be effective in reducing PI. CHX, chlorhexidine; CI, confidence interval; GI, gingival index.

Four studies reported GI data (21–24). CHX demonstrated a statistically significant reduction in GI (mean difference = −0.214; 95% CI: −0.306 to −0.121; 95% PI: −0.532 to −0.105; *P* < .001; I^2^ = 37.3%), indicating low heterogeneity (Figure 5). The greatest GI reductions were observed with 0.2% CHX formulations and rinse or topical product types, whereas lower or higher concentrations showed smaller, non-significant changes. (*P* > 0.05).

**Figure 6:**
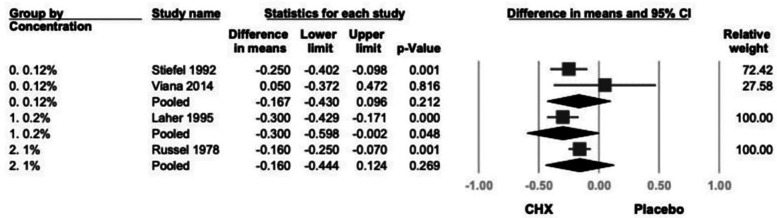
The forest plot of subgroup analysis using concentration of products as the moderator, including 0.2% and 0.12%. CHX is significantly effective in reducing GI in the 0.2% subgroup. CHX, chlorhexidine; CI, confidence interval; GI, gingival index.

Subgroup analysis based on CHX concentration included three groups: 0.12%, 0.2% and 1% (21–24). A significant reduction in GI was observed in the 0.2% CHX group (mean difference = −0.300; 95% CI: −0.598 to −0.002; 95% PI: −1.181 to 0.581; *P* = .048). Although the 0.12% and 1% subgroups showed consistent trends toward GI reduction, the results were not statistically significant. (0.12%: −0.167; 95% CI: −0.430 to 0.096; 95% PI: −0.992 to 0.658; *P* = .212; 1%: −0.160; 95% CI: −0.444 to 0.124; 95% PI: −1.018 to 0.698; *P* = .269) (Figure 6).

**Figure 7:**
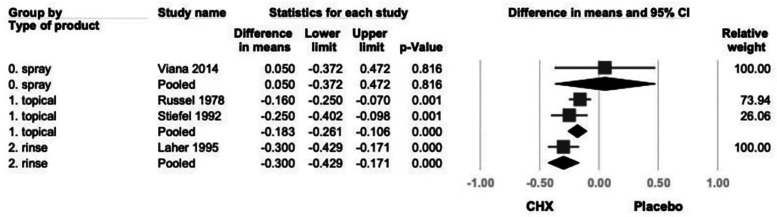
The forest plot of subgroup analysis using concentration of products as the moderator, including 0.2% and 0.12%. CHX is significantly effective in reducing GI in the 0.2% subgroup. CHX, chlorhexidine; CI, confidence interval; GI, gingival index.

Another subgroup analysis based on product type categorized studies into spray, rinse, and topical application (swab or toothpaste) (21–24). While all subgroups demonstrated a consistent trend toward GI reduction, only topical and rinse products reached statistical significance. (topical: −0.183; 95% CI: −0.261 to 0.106; *P* < .001; rinse: −0.300; 95% CI: −0.429 to 0.171; *P* < .001) (Figure 7).

Meta-regression analysis revealed no significant association between treatment duration and GI reduction. (coefficient = −0.0029 per day; *P* = .092) (Supplementary Appendix 5) Visual inspection and Egger's regression test (*P* = 0.829) of the funnel plot showed no evidence of publication bias (Supplementary Appendix 6).

The most commonly used tool was the Löe and Silness GI (*n* = 7) (21–24, 30, 31). A few of the selected studies used bleeding-based indices such as Ainamo and Bay (25), Caton and Polson (27), positive or negative bleeding scores from tooth sites (26) and the remaining studies (29, 32) did not specify the index used.

#### 3.2.3 Bacterial profiles

Oral microflora was reported in 3 studies (28, 30, 32). Two studies stated CHX use was associated with reduction in pathogenic organisms such as spirochetes and motile rods, including an increase in the proportion of cocci (30, 32). Another study stated there was a statistically significant improvement in the composition of the oral microflora 16 weeks after baseline (28).

#### 3.2.4 Complications

Adverse effects were reported in most studies, and were generally mild, localized, and reversible. Tooth staining was the most frequently observed complication, noted in studies using various CHX formulations (21, 22, 25–27). Studies with longer experimental periods (21–31) were more likely to report staining than those with shorter durations (24). One study reported tongue staining and noted difficulty masking the taste of CHX (31). (Table 2).

Complaints about taste and taste disturbances were less common. Two studies reported that participants complained about the flavor of the spray formulation (21, 31). In one study, 4 out of 13 participants in the CHX group complained about the taste (21). Another study found that the participants stated the CHX solution was bitter, resulting in participants at this institution requiring persuasion from ward personnel to continue with its use (31).

Mucosal effects, such as ulceration, were rarely noted. One study observed gingival and buccal mucosa ulceration (29).

Calculus accumulation was reported in two studies, occurring in both intervention and control groups (23, 29).

No serious or irreversible adverse effects were reported in any of the included studies. Two studies reported no complications at all (24, 32).

For this review intervention durations were defined as: short term (2–6 weeks), intermediate term (6–8 weeks) and long term (>8 weeks). This classification reflects the typical duration of use for CHX, and the experimental periods observed in our studies. These findings support the short-term (2–6 weeks) safety of CHX use, though cosmetic and sensory side effects may impact long-term adherence (>8 weeks), particularly in pediatric populations.

### 3.3 Quality assessment and heterogeneity evaluation, quality of evidence

Five studies were rated as low risk in all categories (21, 24–26, 30). The remaining studies showed one or more areas of concern, although none were rated as high risk in any domain (22, 23, 27–29, 31, 32). Overall, the studies included were of sufficient methodological quality to support confidence in our findings (Supplementary Appendix 7). The evidence quality via the GRADE system is moderate.

### 3.4 Sensitivity analysis

Sensitivity analysis via the one-study removal method confirmed the stability of this effect, as no single study significantly altered the overall result for PI (Supplementary Appendix 8), and GI (Supplementary Appendix 9).

## 4 Discussion

This systematic review and meta-analysis aimed to evaluate the effectiveness of CHX in improving oral hygiene and gingival health in patients with SHCN. The findings across the 12 included studies demonstrated that CHX use was consistently associated with reductions in plaque accumulation and gingival inflammation when compared to placebo or mechanical oral hygiene alone. These results were observed across a range of CHX formulations, concentrations, and clinical settings, indicating the robustness of its effect in populations where mechanical plaque removal is often compromised.

The most frequently reported outcomes were PI and GI, both of which showed greater improvement in CHX groups relative to controls. Although outcome measures varied in scale and reporting format, the direction of effect was largely consistent. These findings are particularly relevant in the context of special care dentistry, where patients may face barriers to maintaining oral hygiene due to behavioral, cognitive, or physical limitations. The observed benefits across multiple delivery forms such as rinses, sprays, and gels suggest that CHX can be adapted to meet individual patient needs and clinical circumstances. These results are consistent with prior systematic reviews which have shown similar short-term improvements in plaque control and gingival health following CHX use in other populations (5, 33). This review contributes to the literature by focusing specifically on pediatric and adult populations with SHCN, which are frequently underrepresented in clinical trials despite experiencing a higher burden of oral disease.

In regard to the OM data from 3 studies, it's possible these shifts in the microbial composition of OM may have contributed to the improvements in the plaque and gingival indices observed in these studies (28, 30, 32). Currently evidence is limited regarding the effects of CHX on the OM. Recent reviews suggested OM alteration following the use of CHX mouthwash (34, 35), which may favor the dysbiosis resolution (35). However, the variation in assessing microbial composition in these studies and limited data restricts the ability to create definitive conclusions or infer clinical significance. Recent reviews raise concerns about the risk of bacterial resistance to CHX and accompanying cross-resistance to antibiotics (36–38). In brief, CHX resistance may result from intrinsic resistance from bacterial spores and mycobacteria or extrinsic resistance via the acquisition of mobile genetic elements or gene mutation (37). The emergence of new clones of staphylococci with reduced susceptibility to CHX has been a major concern, especially in immune vulnerable patient populations (39). Also, the use of CHX may also expose microorganisms to sub-inhibitory concentrations of antiseptic in clinical settings. This may create a vicious cycle of resistance, as repeated exposure to sub-inhibitory concentrations can promote an unfavorable environment that facilitates the emergence of new, more resistant microbial clones. Also, the levels of CHX in the selected studies were lower than the concentrations typically used in medical and food service applications (38). Further studies using qualitative and quantitative analyses are required to clarify the impact of CHX on the OM. It also highlights the importance of monitoring changes in CHX resistance and their cross-resistance to antibiotics.

While CHX was generally noted as being well-tolerated, adverse effects were reported in most studies. In the current review, tooth staining was the most common complication, followed by taste alteration and mild mucosal irritation. These effects were typically reversible and self-limiting. Importantly, no studies reported systemic complications or serious adverse events, supporting the short-term safety of CHX when used under appropriate supervision.

From a clinical perspective, these results support the use of CHX as a short-term (2–6 weeks) adjunctive measure in populations with reduced ability to maintain mechanical plaque control. CHX use was associated with meaningful improvements in gingival health across studies. Subgroup analyses showed that 0.2% CHX formulations provided the greatest benefit. Additionally, longer treatment durations were associated with greater plaque improvements. However, adverse effects such as tooth staining, taste alteration, and mucosal irritation were frequently reported, even in studies lasting just 4–8 weeks. These effects became more pronounced in studies with longer duration.

Based on these findings, CHX appears most effective and tolerable when used short-term, between 2 and 6 weeks, particularly in individuals with limited ability to perform mechanical plaque control. Intermediate use, between 6 and 8 weeks, may improve outcomes but should be weighed against the increased risk of adverse effects. Long-term use, beyond 8 weeks, in patients with SHCN cannot be recommended unless carefully monitored by a dental provider.

Future research should aim to standardize outcome measures, stratify patient subgroups more clearly, and explore the long-term effects of CHX use in pediatric and adult populations with SHCN. Studies incorporating microbiological outcomes and patient-reported measures, such as acceptability and adherence, would also enhance the clinical relevance and applicability of future findings.

Several limitations should be acknowledged. The included studies were heterogeneous in terms of intervention duration, CHX concentration and formula, delivery method, and outcome assessment, such as the variation in clinical indices used. Thus, we standardized measurement using Hedges' *g*. tThe random-effects model and subgroup analysis were used to address this heterogeneity, following Cochrane Handbook guidelines. Differences in intervention duration across trials could have affected effect estimates, so meta-regressions were conducted to assess linear relationships between these factors and index reduction (15, 19, 20, 40). Additionally, some studies did not specify the exact plaque or gingival indices used, limiting comparability across trials. Also, including only English language studies, due to a lack of high-quality translation resources, may be source of bias in the current study. It should be noted that most of selected studies had short experimental periods, which prevents further investigation on the long-term efficacy and safety of CHX use. Limited data are available to establish the relationship between OM and CHX use, or to address concerns regarding CHX resistance in patients with SHCN. Finally, definitions of SHCN varied, and in some cases were not clearly described, which may affect the generalizability of findings. The inclusive nature of the SHCN definition allowed for a broad catchment of studies; however, this also introduced heterogeneity in the study populations. While most studies clearly identified participants with intellectual or developmental disabilities, a few used broader or less specific terms such as “special needs” without detailed diagnostic criteria. This variability may affect the internal validity of pooled data and limit the generalizability of findings. Nonetheless, individuals with SHCN—regardless of specific diagnosis—often face similar clinical challenges, including reduced dexterity for oral hygiene, medication-induced salivary changes, and behavioral limitations that complicate routine dental care. In general, the overall bias of the selected articles was low to some concerns. However, it is noteworthy that the PI may present potential publication bias due to the small study effects and abovementioned heterogeneity. Future research would benefit from more standardized reporting of participant characteristics to enhance comparability across studies.

## 5 Conclusion

CHX demonstrates consistent effectiveness in reducing plaque accumulation and gingival inflammation among patients with SHCN, particularly in individuals with limited dexterity to perform mechanical plaque control. Short-term use (2–6 weeks), especially with 0.2% formulations, appeared to offer the greatest benefit while minimizing side effects. Although adverse effects such as tooth staining and taste alterations are common, they are generally mild and self-limiting. These findings support the short-term use of CHX as an adjunctive measure in oral hygiene management for individuals with SHCN, with use tailored to the patient's needs and monitored closely by a dental provider to minimize complications. Further research should explore the long-term effects of CHX in patients with SHCN, including changes to OM and adverse impacts of treatment.

## Data Availability

The original contributions presented in the study are included in the article/Supplementary Material, further inquiries can be directed to the corresponding author.
