# Supplementary material for: Does chlorhexidine improve periodontal health and bacterial profiles in patients with special health care needs? A systematic review and meta-analysis
Source: Front Oral Health. 2025 Nov 18;6:1656328. doi: 10.3389/froh.2025.1656328 (PMC12669217; doi:10.3389/froh.2025.1656328)

**Does Chlorhexidine Improve Periodontal Health and Bacterial Profiles in Patients** 1 **with Special Health Care Needs? A Systematic Review and Meta-Analysis**

Appendices **(from RE)**

Appendix 1. Search Strategies

Appendix 2. PRISMA flow chart

Appendix 3. Excluded articles and reasons

Appendix 4. Funnel plot of the included trials showed some asymmetry in effect size distributions in articles measuring plaque index

Appendix 5. Meta-regression of difference in means on treatment duration (day).

Appendix 6. Funnel plot of the included trials showed no evidence of publication bias in articles measuring gingival index.

Appendix 7. Quality Assessment

Appendix 8. Results of a sensitivity analysis using the one-study removal method for plaque index (PI)

Appendix 9. Results of a sensitivity analysis using the one-study removal

method for gingival index (GI).

Appendix 1. Search Strategies (see the descriptions, no figures)

Appendix 2. PRISMA flow chart


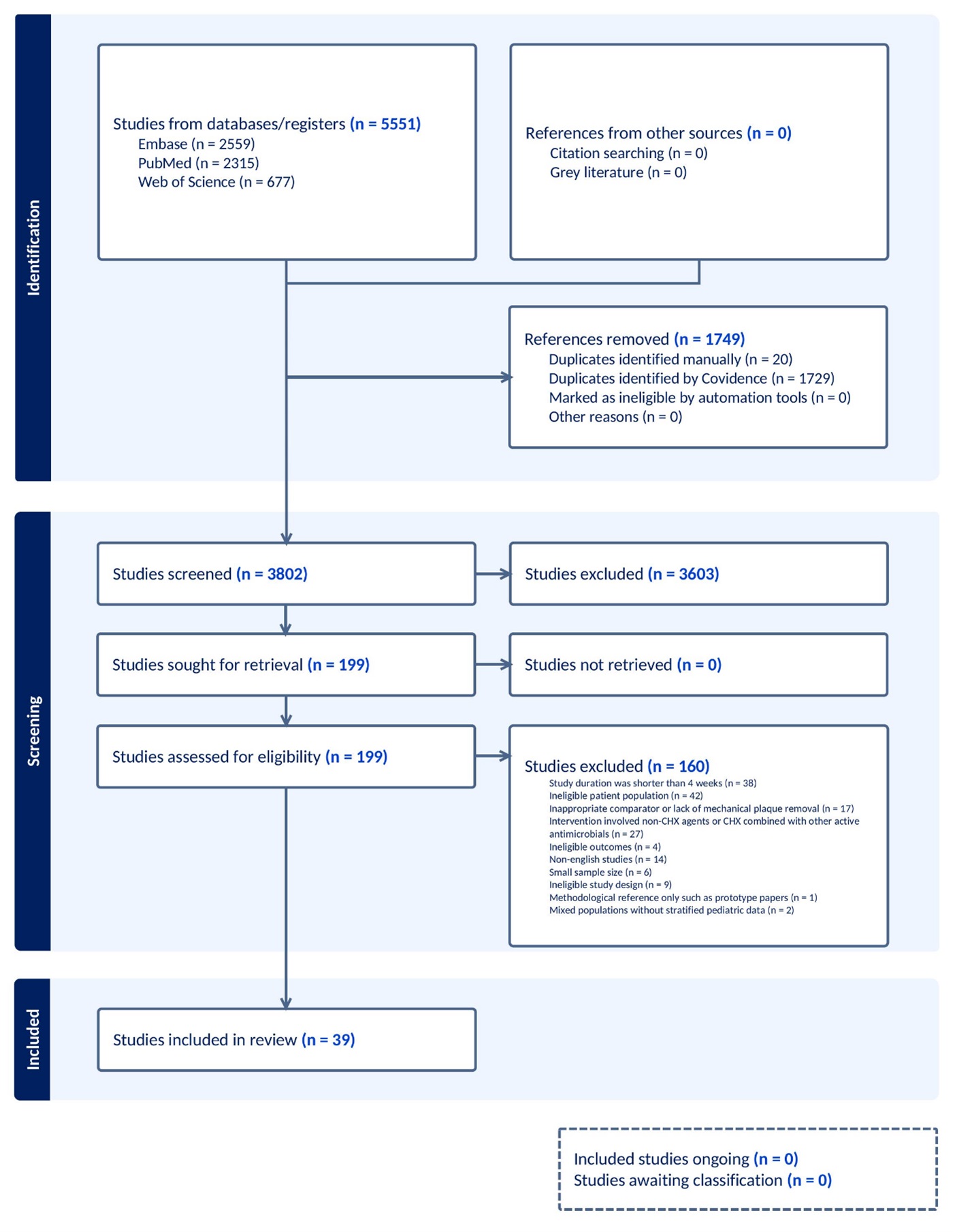


Appendix 3. Excluded articles and reasons (see the table)

Appendix 4. Funnel plot of the included trials showed some asymmetry in effect size distributions in articles measuring plaque index


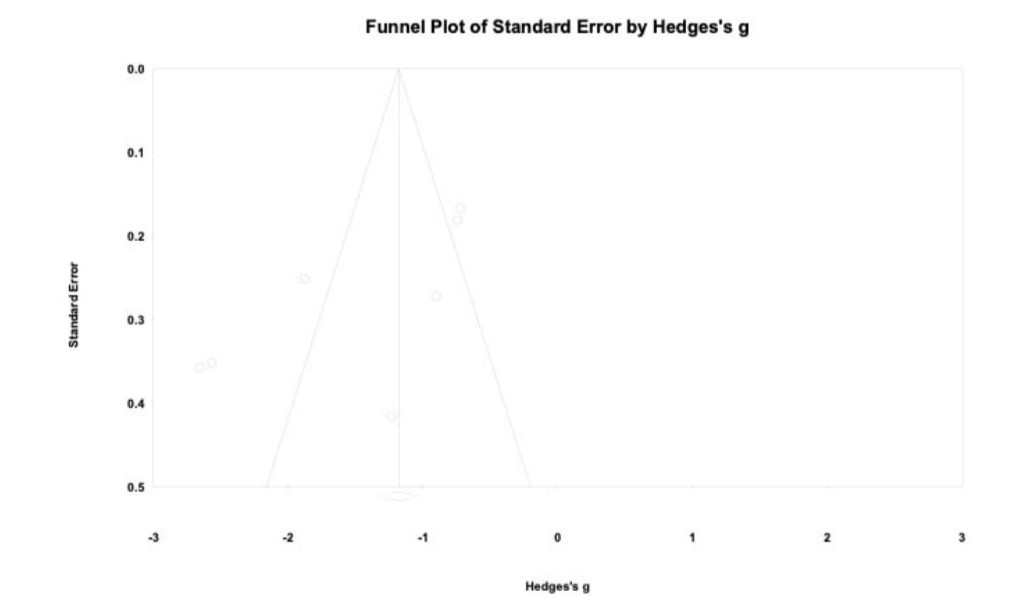


Appendix 5. Meta-regression of difference in means on treatment duration (day).


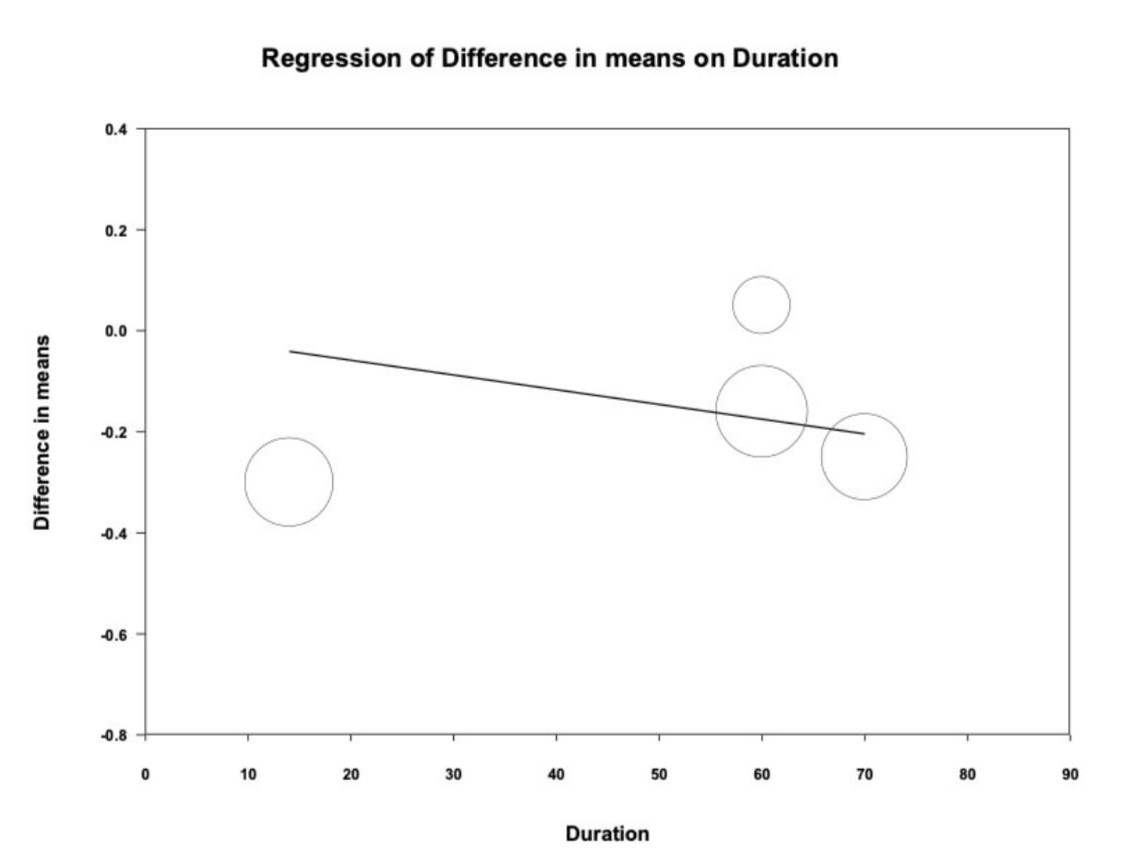


Appendix 6. Funnel plot of the included trials showed no evidence of publication bias in articles measuring gingival index.


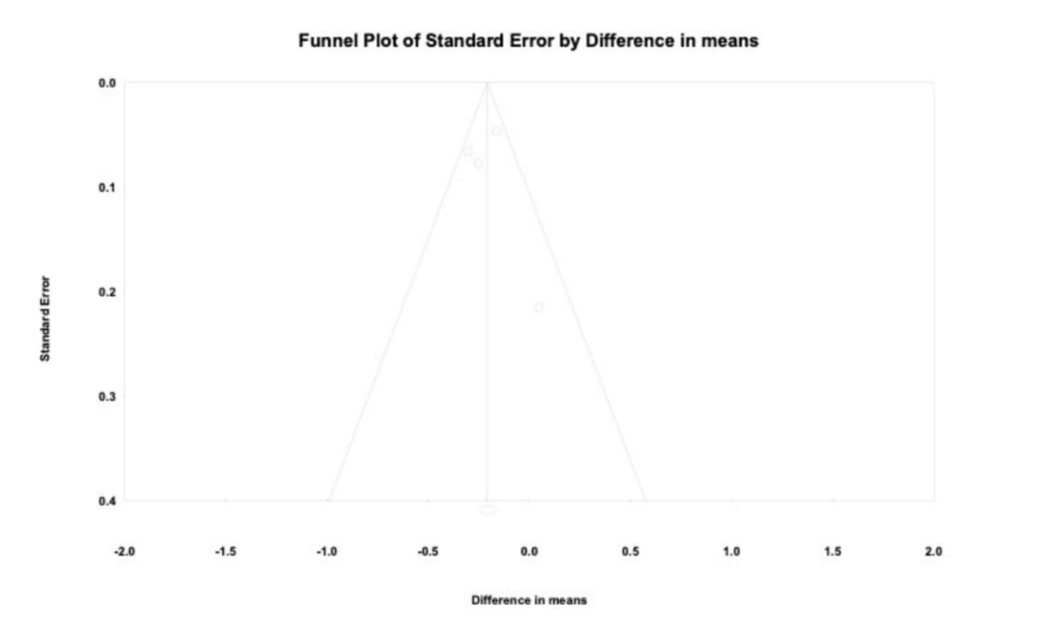


Appendix 7. Quality Assessment


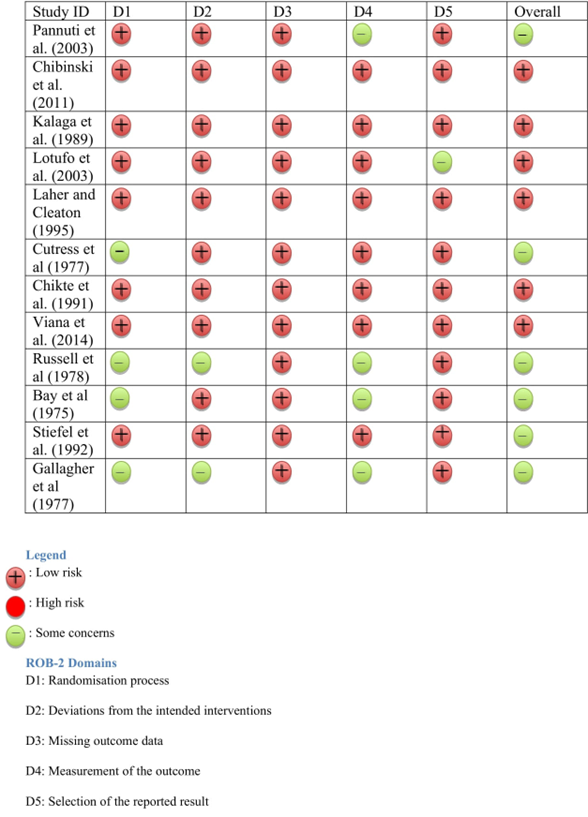


Appendix 8. Results of a sensitivity analysis using the one-study removal method for plaque index (PI)


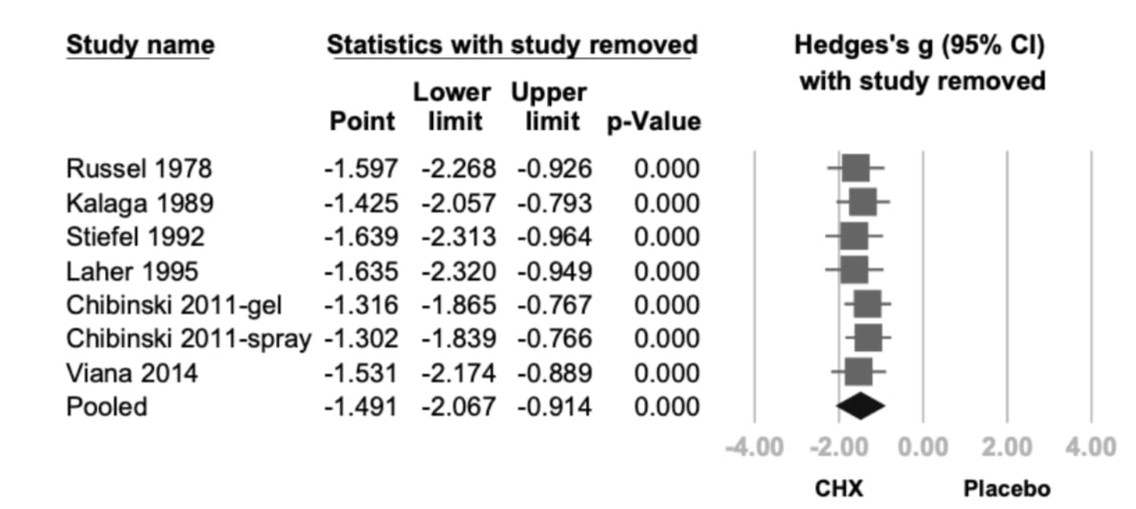


Appendix 9. Results of a sensitivity analysis using the one-study removal method for gingival index (GI).


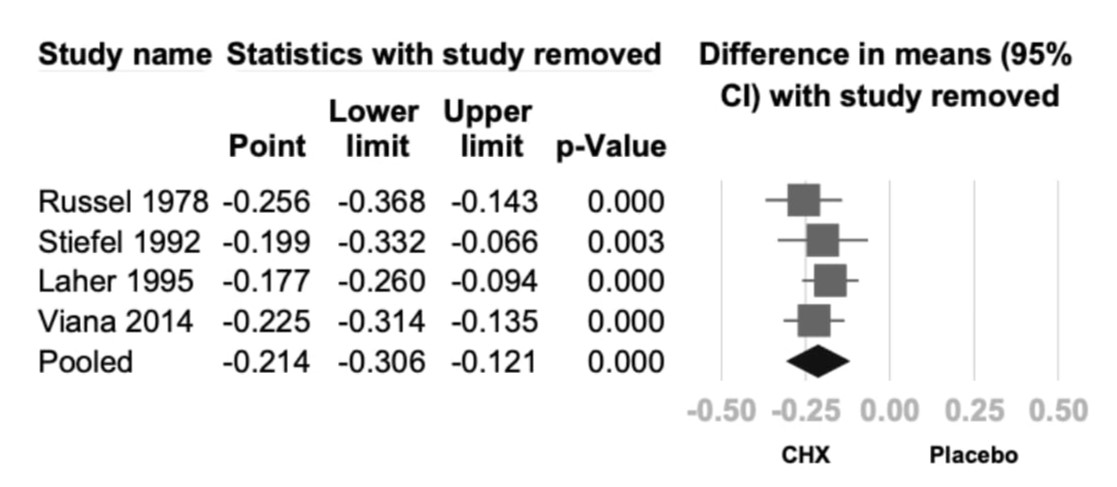

Supplement: Supplementary file 1 [file Datasheet1.docx]
